# Gender differences in the prevalence of mental distress in East and West Germany over time: a hierarchical age-period-cohort analysis, 2006–2021

**DOI:** 10.1007/s00127-023-02479-z

**Published:** 2023-04-11

**Authors:** Daniëlle Otten, Ayline Heller, Peter Schmidt, Manfred E. Beutel, Elmar Brähler

**Affiliations:** 1 Department of Psychosomatic Medicine and Psychotherapy, University Medical Center of the Johannes Gutenberg-University Mainz, Langenbeckstraße 1, 55131 Mainz, Germany; 2 Department of Psychiatry and Psychotherapy, Leipzig University Medical Center, Leipzig, Germany

**Keywords:** Mental distress, Age-period-cohort, Temporal trends, Gender, East and West Germany

## Abstract

**Purpose:**

Mental distress has become a major public health concern. Temporal trends in psychological distress are complex and depend on numerous factors. In this study, we examined age-period-cohort effects for mental distress including gender and German region over a 15 years’ time span.

**Methods:**

Data on mental distress from ten cross-sectional surveys of the general German population, covering the years from 2006 to 2021, was used. Hierarchical age-period-cohort analyses including gender and German region as predictors were performed to disentangle age, period, and cohort effects. The Patient Health Questionnaire-4 was used as a brief screener for mental distress.

**Results:**

We found significant period and cohort effects, with peek values for mental distress in the years 2017 and 2020 and for the oldest birth cohort (born before 1946). Age did not affect mental distress when cohort- and period effects as well as gender and German region were considered. An interaction effect for gender and the German region was found. Women in West Germany reported significantly higher mental distress compared to women in East Germany. Compared to men, women reported the highest prevalence in both regions.

**Conclusion:**

Important political events as well as major crises can lead to an increase of mental distress in societies. Furthermore, an association between birth cohort and mental distress could be linked to socialization effects of that certain time, causing traumatic experiences or a specific coping style within this cohort group. Prevention and intervention strategies could benefit from acknowledging structural differences linked to period and cohort effects.

**Supplementary Information:**

The online version contains supplementary material available at 10.1007/s00127-023-02479-z.

## Introduction

Mental health, a state of wellbeing that allows the individual to cope with stressors of everyday life and function productively [1], is characterized to a large part by the absence of mental distress and mental disorders. Both mental distress and mental disorders have become major public health concerns affecting the quality of life, work productivity, physical illnesses, and life expectancy of a large proportion of the general population [2]. While mental distress refers to distress in response to an external stressor and can be characterized by e.g., (symptoms of) anxiety or depression [3], psychological disorders consist of a pattern of persistent behavioral or psychological symptoms that influence several areas of life.

Mental health is subject to different temporal trends: it may vary depending on age, time period, and birth cohort. Moreover, mental health differences are frequently found between women and men, and between regions. When analyzing temporal trends, it is important to note that age, period, and cohort effects are highly related to one another. Age effects refer to developmental or age-specific transformation, the general pattern of individual transformation from childhood through adulthood and old age that are consistently noted in all birth cohorts and across all time periods [4]. Period effects, on the other hand, describe differences among individuals due to historical events that leave unique imprints, no matter the age. Finally, cohort effects refer to differences among individuals categorized by their time of birth, they share critical formative moments and similar socialization experiences with their respective birth cohort [4]. Ideally, age, period, and cohort effects are examined simultaneously. However, due to the exact multicollinearity and conceptual relationship between age, period, and cohort, it is difficult to correctly estimate these effects. Studies apply different methodical frameworks based on specific theory-based assumptions to disentangle these effects [5]. Thus, comparisons between studies are feasible only to a limited extent.

With regard to age effects, a Dutch study revealed self-reported prevalence of mental illnesses to be lower and general mental health to be better for the elderly [6]. Similar results are found in a study in the USA; using the Composite International Diagnostic Interview (CIDI), lifetime and recent major depressive episodes are less prevalent among respondents of 65 years and older [7]. However, a longitudinal survey study covering a 15-year period found a U shape for depressive symptoms with the highest symptoms burden for the age group 25–35 years and from 75 years onwards after controlling for cohort effects [8]. A study including 27 European countries assessed self-reported depressive symptoms and also found the highest prevalence of the current depressive disorder among persons 75 years and older [9]. A hierarchical age-period-cohort analysis (HAPC) on the life course trajectory of mental health from the UK partly confirmed this finding, as it revealed mental health increases throughout the life-course, but slows during middle-age and worsening again slightly in older age [10]. In Germany, the prevalence of current depressive symptoms (self-reported) was highest among 18–29-year-olds and decreased with age, whereas the lifetime prevalence of diagnosed depression was highest among 60–69-year-olds [11]. Among the German elderly (53–80 years), a U shape for the prevalence of depressive symptoms was found [12].

Period and cohort effects are also found in mental health studies. In a study applying HAPC models, recent birth cohorts in the UK generally reported worse mental health [10]. A study from 1993 examining age and cohort effects for the occurrence of depression in a US sample reported that the birth cohort 1950–1959 had the lowest age of a first episode of depression [13]. An age-period-cohort (APC) analysis in Canada and USA revealed the highest levels of psychological distress in the oldest (born before 1939) and more recent (born 1989–1992) birth cohorts [14]. With regard to period effects, a systematic review and meta-analysis addressing the period 1980–2013 reported the highest prevalence estimates of common mental disorders worldwide in studies undertaken in the 1990s [15]. In Canada and the USA, levels of mental distress were highest around 2000 [14]. In the USA, reported depressive symptoms were highest between 2000 and 2010 [16]. A German study applying HAPC models to examine age-period-cohort trends in depressive symptoms found a U-shaped cohort effect where cohorts born around 1930 until 1950 exhibited less depressive symptoms compared to earlier and later-born cohorts [17].

Within Germany, differences in mental health are found between the former eastern and western federal states. Founded after World War II (WWII), the two German States existed from 1949 to 1990. They evolved with contrary and antagonistic political and economic systems. The federal republic of Germany followed the (capitalist) system of the Western European countries, whereas the German Democratic Republic (GDR) followed the example of the Russian-Soviet (socialist) system. The socialization processes and living conditions were therefore extremely different between the former East and West Germany, leading to different risk and protective factors regarding mental health. While many people suffered from political persecution and repression, leading to increased somatic symptoms, anxiety and depression later in life [18], other system-related factors like a low official unemployment rate and increased social mobility could be regarded as protective factors for mental health. The re-unification was accompanied by drastic changes in almost all aspects of life; while average income has been increasing in East Germany since 1990, they remain lower compared to West Germany [19, 20], and the unemployment rate 20 years after re-unification was still twice as high in East compared to West Germany [21]. Regarding demographic characteristics, the East German population was reduced by 16% since re-unification [19], especially young people and women left East Germany. Even though the inner German migration has aligned now, the East still consists of an older population strata [19] and has a lower life expectancy than West Germany. These sociodemographic and socioeconomic differences are reflected in birth cohorts.

With regard to mental health, evidence shows that results for East and West Germany strongly differ between survey year and mental health outcome. A study examining psychological distress and mental disorders in former East and West Berlin one year after the fall of the Berlin wall did not find differences in ICD-10 diagnoses [22]. However, a large nationally representative survey reported higher prevalences of mental disorders (assessed with CIDI) in the Western compared to the Eastern states in 1998/1999 [23] concerning depression, somatoform disorder, substance abuse, eating disorders, and social anxiety. Studies comparing mental health between East and West Germany 10 years after reunification found no differences in mental health between participants residing in East and West Germany [24], whereas life satisfaction was higher among West Germans compared to East Germans [25]. Perceived stress did not differ between formerly East and West Germany 20 years after reunification [26], whereas the prevalence of depression diagnosis was found to be lower in East compared to West Germany [27]. Another study, however, did not confirm these differences for adolescents [28]. Evidence for mental health differences between East and West Germany is thus inconclusive. An analysis covering a longer time frame and using representative data from Germany is thus needed to shed further light on the temporal trends in these two regions.

Differences in mental health exist between women and men. For most internalizing disorders (e.g., major depression [29] and anxiety disorders), women are more frequently affected than men [30, 31], whereas for externalizing disorders (e.g., substance abuse) men are more frequently affected [32]. Representative German studies have shown that approximately one in three women and one in four or five men had a diagnosis of a mental disorder in the previous 12 months [33]. Sex differences in mental health can be explained by hormones [34, 35] and dysregulations in the hypothalamic–pituitary–adrenal (HPA) axis [34, 36], especially for stress-related mental disorders. Gender differences in mental health can be explained by e.g., gender-based violence [37], low self-esteem [34], and differences in risk behavior and identification of disease symptoms [38]. Hence, sex and gender interact in the development of diseases [39, 40]. Myocardial infarction and depression are gender-stereotypical diseases [41]. Myocardial infarction is known as a typical disease for men and is therefore often overlooked for women [40], while depressive disorders are considered a typical disease for women and underdiagnosed for men [41].

Age effects in mental distress differ for women and men. Results from an Australian study reveal a consistent decrease in mental distress for women, whereas for men, the decrease only starts in late adulthood [42]. In the USA, women in all age groups report depression more often than men and this gender gap increases in adulthood [43]. Regarding period effects, a Swedish study showed that the prevalence of self-reported anxiety increased between 1980/81 and 2004/5 for women and men in most age groups, except for men aged 64–71 and women aged 56–63 [44]. Additionally, cohort effects were found; for men, anxiety increased from birth cohort 1942–40 onwards, while for women, this increase was already observed from birth cohort 1926–33 and stagnated with birth cohort 1974–81 [44]. With regard to period trends, a British study shows increased mental distress especially for women between the years of 1991 and 2008 [45]. Moreover, an Australian study revealed increased mental distress between 2001 and 2017 for both women and men [46]. The prevalence of diagnosed depression increased in Germany between 2009 and 2017, especially in young men [47]. Once again, none of these studies estimated age, period, and cohort effects simultaneously with regard to differences between men and women.

## Objective of this study

To gain more insight into temporal trends of self-reported psychological distress in East and West Germany and between women and men, we will apply HAPC analyses. In these analyses, cohort and period are modelled as random effects and age, gender, region, and control variables are modelled as fixed effects. The present work aims to disentangle the effects of mental distress caused by different political system. The following two main research questions are studied:

(I) Are age, period, or cohort associated with mental distress in the years from 2006 until 2021?
(II) What role do gender and German region play as main predictor variables within these associations?

## Methods

### Sample

Data from ten German representative studies with the same recruitment procedure conducted in the years 2006, 2010, 2014, 2017, 2018, 2019, 2020, and 2021 were analyzed. Sample sizes ranged from 2503 participants in 2020 to 5036 participants in 2006 (Supplementary Table S1). Data were collected by an independent agency (USUMA, Berlin) in nationwide surveys. Samples were representative in terms of age, gender and education. Applied eligibility criteria were an age of at least 14 years and a sufficient understanding of the German language. Participants were chosen via a random-route procedure. Individuals in multi-person households were randomly selected using a Kish Selection Grid. The target person participated in a face-to-face interview conducted by a trained interviewer and additionally independently filled out several questionnaires. See Supplementary Table S1 for further information on each survey.

Before interviews started, all potential participants were informed of the aims of the respective survey that included aspects of general and mental health as well as political attitudes and beliefs, method of data collection, and handling of data including data privacy and anonymity in responses. They then provided informed consent. Minors gave informed assent and informed consent was given by their parents or legal guardians. The study and procedure, including the consent procedure, were approved by the institutional ethics review board of the University of Leipzig (s. ethic approval numbers in Supplementary Table S1).

For this study, we excluded participants who did not fully complete the mental distress questionnaire, e.g. participants with missing values on at least one of the items (*n* = 290, 1.05%). Since these missings did not exceed 5%, they were deleted listwise [48]. Furthermore, we excluded people with gender ‘divers’ (*n* = 5), since it was not possible to compute analyses on this very small group. Lastly, we selected participants of 16 years and older, yielding a final sample of 27,033 individuals. The sample consisted of 14,560 (53.9%) women and 12,473 (46.1%) men with 5445 persons living in East Germany (20.1%) and 21,588 in West Germany (79.9%). Women were overrepresented in both East and West Germany (52.3% versus 47.7% in East Germany; 54.2% versus 45.8% in West Germany). Respondents showed an age range of 16 to 99 years (*M* = 49.2; SD = 17.7). For a sample description per included study year, see Table 1.

**Table 1:** Description of complete sample (*N* = 27,033) stratified by gender and German region

Survey year	*N*	Gender		Region		Age	Birth cohort					Partner		Nett household income		
		Men (%)	Women (%)	West Germans (%)	East Germans		< 1946 (%)	1946–1959 (%)	1960–1969 (%)	1970–1980 (%)	> 1980 (%)	Not living together (%)	Living together (%)	< 1250 (%)	1250–2500 (%)	From 2500 onwards (%)
Total	27,033	12,473 (46.1)	14,560 (53.9)	21,588 (79.9)	5445 (20.1)	49.20 ± 17.70	4491 (16.6)	6188 (22.9)	5388 (19.9)	4580 (16.9)	6386 (23.6)	14,217 (52.6)	12,761 (47.2)	4802 (17.8)	11,770 (43.5)	9646 (35.7)
2006	4916	2266 (46.1)	2650 (53.9)	3927 (79.9)	989 (20.1)	48.90 ± 17.55	1513 (30.8)	1163 (23.7)	962 (19.6)	778 (15.8)	500 (10.2)	2235 (45.5)	2681 (54.5)	1058 (21.5)	2564 (52.2)	1045 (21.3)
2010	2458	1133 (46.1)	1325 (53.9)	1962 (79.8)	496 (20.2)	51.16 ± 18.09	690 (28.1)	576 (23.4)	439 (17.9)	388 (15.8)	365 (14.8)	1184 (48.2)	1274 (51.8)	508 (20.7)	1276 (51.9)	602 (24.5)
2013	2441	1139 (46.7)	1302 (53.3)	1946 (79.7)	495 (20.3)	50.08 ± 17.90	473 (19.4)	610 (25.0)	447 (18.3)	398 (16.3)	513 (21.0)	1344 (55.1)	1097 (44.9)	506 (20.7)	1118 (45.8)	744 (30.5)
2014	2459	1133 (46.1)	1326 (53.9)	1965 (79.8)	494 (20.1)	50.01 ± 17.36	403 (16.4)	630 (25.6)	478 (19.4)	428 (17.4)	520 (21.1)	1304 (53.0)	1155 (47.0)	454 (18.5)	1054 (42.9)	888 (36.1)
2016	2406	1089 (45.3)	1317 (54.7)	1915 (79.6)	491 (20.4)	49.47 ± 17.69	323 (13.4)	556 (23.1)	503 (20.9)	403 (16.7)	621 (25.8)	1095 (45.5)	1280 (53.2%)	444 (18.5)	1013 (42.1)	876 (36.4)
2017	2483	1111 (44.7)	1372 (55.3)	1994 (80.3)	489 (19.7)	49.08 ± 17.59	284 (11.4)	575 (23.2)	518 (20.9)	410 (16.5)	696 (28.0)	1414 (56.9)	1069 (43.1%)	380 (15.3)	1027 (41.4)	1005 (40.5)
2018	2477	1125 (45.4)	1352 (54.6)	1980 (79.9)	497 (20.1)	48.33 ± 17.38	226 (9.1)	545 (22.0)	522 (21.1)	469 (18.9)	715 28.9)	1404 (56.7)	1067 (43.1%)	414 (16.7)	1020 (41.2)	1043 (42.1)
2019	2448	1143 (46.7)	1305 (53.3)	1959 (80.0)	489 (20.0)	48.97 ± 17.48	215 (8.8%)	556 (22.7)	493 (20.1)	454 (18.5)	730 (29.8)	1351 (55.2)	1089 (44.5%)	328 (13.4)	976 (39.9)	1098 (44.9)
2020	2442	1146 (46.9)	1296 (53.1)	1951 (79.9)	491 (20.1)	46.37 ± 17.51	144 (5.9)	446 (18.3)	525 (21.5)	415 (17.0)	912 (37.3)	1459 (59.7)	975 (39.9%)	331 (13.6)	754 (30.9)	1189 (48.7)
2021	2503	1188 (47.5)	1315 (52.5)	1989 (79.5)	514 (20.5)	50.26 ± 18.03	220 (8.8)	531 (21.2)	501 (20.0)	437 (17.5)	814 (32.5)	1427 (57.0)	1074 (43.0)	379 (15.1)	968 (38.7)	1156 (46.2)

For the year 2017, sample recruitment took place between November 2017 and January 2018. The vast majority were recruited in 2017 (80%). For the year 2021, sample recruitment took place between December 2020 and January 2021. The vast majority were recruited in 2021 (85%). Descriptive statistics were performed as absolute and relative proportions for categorical data and means and standard deviations (M ± SD) for continuous variables

### Measures

The Patient Health Questionnaire-4 (PHQ-4) is an ultra-brief reliable and valid screener for depression and anxiety [49]. An update of normative data from the German general population reports acceptable reliability for PHQ-4 based on McDonald’s omega (*ω* = 0.85; 95% CI 0.84–0.86) [50]. Depression consists of two items of the screening instrument Patient Health Questionnaire-9 (PHQ-9) [51], namely: “little interest or pleasure in doing things” and “feeling down, depressed, or hopeless”. Anxiety includes the two screening items of the Generalized Anxiety Disorder-7 (GAD-7) [52]: “Feeling nervous, anxious or on edge” and “not being able to stop or control worrying”. The frequency of occurrence in the past two weeks was rated from 0 = “not at all”, 1 = “several days”, 2 = “over half the days”, and 3 = “nearly every day” for all items. The sum score of the four items was calculated as a measure of mental distress (range 0–12). A higher score indicated more mental distress. For the analyses, a single-factor solution was used as a measure of distress.

Definitions of generations differ between East and West Germany. For East Germany, the grouping of Ahbe and Gries [53] is often applied, whereas for West Germany the division of Klimczuk [54] is more suitable. To create cohorts that represent both East and West German, these two definitions were combined and birth cohorts were divided into five groups. The first group existed of respondents born before 1946 and was labelled as pre- WW II/WW II generation. The second group included the birth years 1946 until 1959 and represented the post-war generation experiencing the formation of the two separate German states, the beginning of the cold war, and the economic growth in the west. Birth cohorts in the third group from 1960 to 1969 were labelled the cold war area generation. The fourth group included birth cohorts from 1970 until 1980 representing respondents being children at the time of the existence of the former Democratic Republic but experiencing unified Germany as adults. The fifth and last group included respondents born after 1980. This group had little to no experience with the separate states but did experience the transformation in the east. In conclusion, each of the five cohort groups (born: < 1946, 1946–1959, 1960–1969, 1970–1980, > 1980) contained an approximately equal number of participants.

Age and survey year were included as continuous variables. Gender (1 = men, 2 = women) and region (1 = West Germany, 2 = East Germany) were included as main predictor variables. Living with partner (0 = no, 1 = yes) and categorical net household income (< 1250, 1250–2500 and from 2500 onwards) were included as confounders.

### Statistical analyses

All analyses were performed in R version 3.6.3. First, descriptive analyses were computed to provide information on differences in the prevalence of mental distress between gender and region.

Afterwards, multi-group confirmatory factor analysis (MGCFA) was applied for age groups, birth cohorts and survey years to test the measurement invariance (MI) of the PHQ-4. In these MGCFA’s, three models were tested sequentially, with each level introducing an additional restriction to the model. The configural, metric, and scalar model test invariance for the factor structure, factor loadings, and intercept values between groups. MI testing included a series of model comparisons by applying adjusted *χ*^2^-difference tests [55]. A non-significant *χ*^2^-difference (*p* ≥ 0.010) indicates MI among the tested models. As the *χ*^2^-statistic is sensitive to sample size, we further focused on the differences ΔCFI; values ≤ 0.01 indicate the invariance of the models [56, 57]. Besides ΔCFI, we examined the standardized root mean square (SRMR), which is an absolute measure of fit. A value less than 0.08 is considered as a good model fit [58]. The root mean square error of approximation (RMSEA) is often used as goodness-of-model fit. However, the RMSEA often falsely indicates a poor fitting model when models with small degrees of freedom are tested [59], and was therefore not reported.

To test whether age, birth cohort and time period affect mental distress, HAPC analyses were conducted. APC analyses are impeded by the perfect multicollinearity between age, period, and birth cohort: any two of the three dimensions, age, period, and cohort, fix the third. Yang and Land [60] offer a solution to this problem using multilevel modelling on repeated cross-sectional sample survey data: age and age^2^ are included as fixed effects, whereas cohort and period are included as random effects. The APC models were fitted using the *lmer* function within the *lme4* package in *R*. In our first model (M1), age, age^2^, cohort, and period were tested. In the second model (M2), the main predictor variables gender and German region were added to the model. The third model (M3) additionally included control variables and in the last model (M4), an interaction term for gender with German region was implemented. For all models, marginal and conditional *R*^2^ for explained variance was reported. To assess the significance of period and cohort effects, the fit of models without each of these terms (i.e., models A + C and A + P) were compared with the fit of the complete model (A + P + C) [61, 62].

To allow for simultaneous estimation of the APC effects, strong assumptions about the nature of the data have to be made that cannot be tested directly [5]. We, therefore, performed several robustness tests. Firstly, estimates from the hierarchical age-period-cohort models with unequal intervals for age, year, and cohort may depend on the width chosen for these intervals [63]. We thus tested our first model (M1) using different grouping variables of birth cohorts and compared them. Secondly, the HAPC method could be biased, i.e. results may be a consequence of data structure [64], especially when it comes to near-linear trends in the random part of the model. In our case, this means linear trends in cohort and period effects would be underestimated or ignored. We thus compared our first model (M1) with other model variants. To test for linear effects of cohort or period, we subsequently moved one of them from the random part of the model into the fixed part of the model while the other one remained a random factor. We then compared the new models with M1. Furthermore, to examine both a linear and non-linear effect of cohort and period, we compared our main model (M1) with models including cohort or period in both the fixed and random part of the model respectively. We compared Akaike Information Criterion (AIC), Bayesian Information Criterion (BIC), logLikelihood, and Deviance to determine which model fit the data better.

## Results

### Descriptive analyses

The prevalence of mental distress fluctuates over time. The highest levels of mental distress were reported in 2020 (*M* = 2.18, SD = 2.31) and the lowest in 2016 (*M* = 1.42, SD = 2.18) and 2021 (*M* = 1.41, SD = 2.10), see Supplementary Table S2a. Moreover, prevalence rates of mental distress differ across age groups. The highest levels of mental distress were found for the oldest age group, namely 75 years and older (*M* = 2.24, SD = 2.47). Lowest levels of mental distress were reported by respondents in the age groups 25 to 34 (*M* = 1.58, SD = 2.12) and 35 to 44 (*M* = 1.55, SD = 2.14), see Supplementary Table S2b. Finally, the prevalence of mental distress differs across cohorts with the oldest cohort, born before 1946, reporting the highest levels of mental distress (*M* = 1.99, SD = 2.33), see Supplementary Table S2c.

**Fig. 1:**
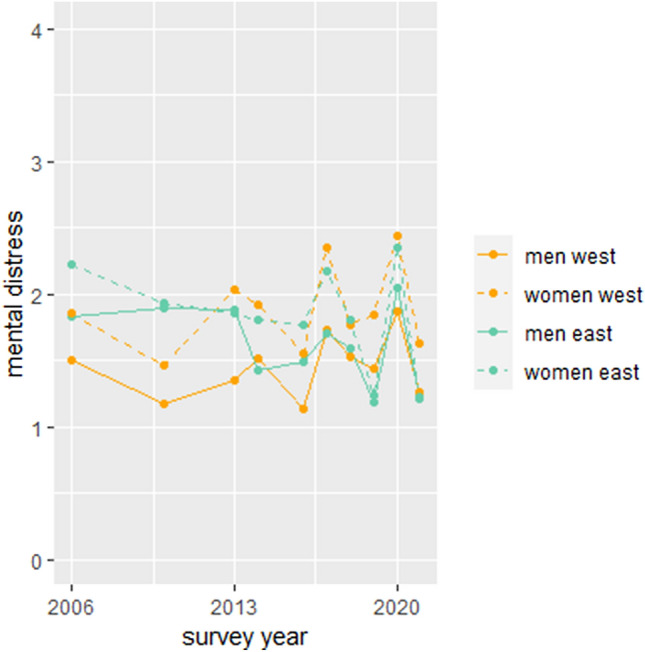
Mental distress over time by gender and German region. Mean values of mental distress are reported

When comparing women and men as well as East and West Germany, results reveal that throughout the years, average levels of mental distress have always been higher for women compared to men. However, the pattern of levels of distress over time was similar for women and men. Differences in levels of mental distress between East and West Germany were found in 2006 and 2010 with higher levels of mental distress for East Germans (s. Supplementary Table S2a). Hereafter, alternating higher levels of distress were found in East and West Germany. In the last year, levels of mental distress were higher for West Germans. The course of mental distress for women and men in East and West Germany over time is displayed in Fig. 1. With regard to age groups, women in all age groups reported higher mental distress compared to men, but the course was similar for women and men. The same applies to gender differences in mental distress across cohorts. Women reported more mental distress than men within each cohort, however, the patterns of levels of distress were similar. No significant differences between East and West Germany across age groups and cohorts were found (s. Supplementary Tables S2b and S2c).

### MI PHQ-4 for age groups, birth cohorts and survey year

To ensure that mean values of PHQ-4 can indeed be compared between survey years, birth cohorts and age groups, we performed MGCFA to test MI. The results are shown in Supplementary Table S3. For the MGCFA including survey year, the configural model had a good model fit (CFI = 0.98, SRMR = 0.02). The changes in CFI in the metric compared to the configural model and the scalar compared to the metric model were all smaller than 0.01. The value of SRMR remained far below 0.08. This indicated that factor structures, factor loadings and intercept values are similar across survey years and mean values and regressions coefficients for PHQ-4 can be compared across survey years. The MGCFAs including birth cohort and age groups also revealed measurement invariance for PHQ-4 across the respective groups.

### APC analyses

**Fig. 2:**
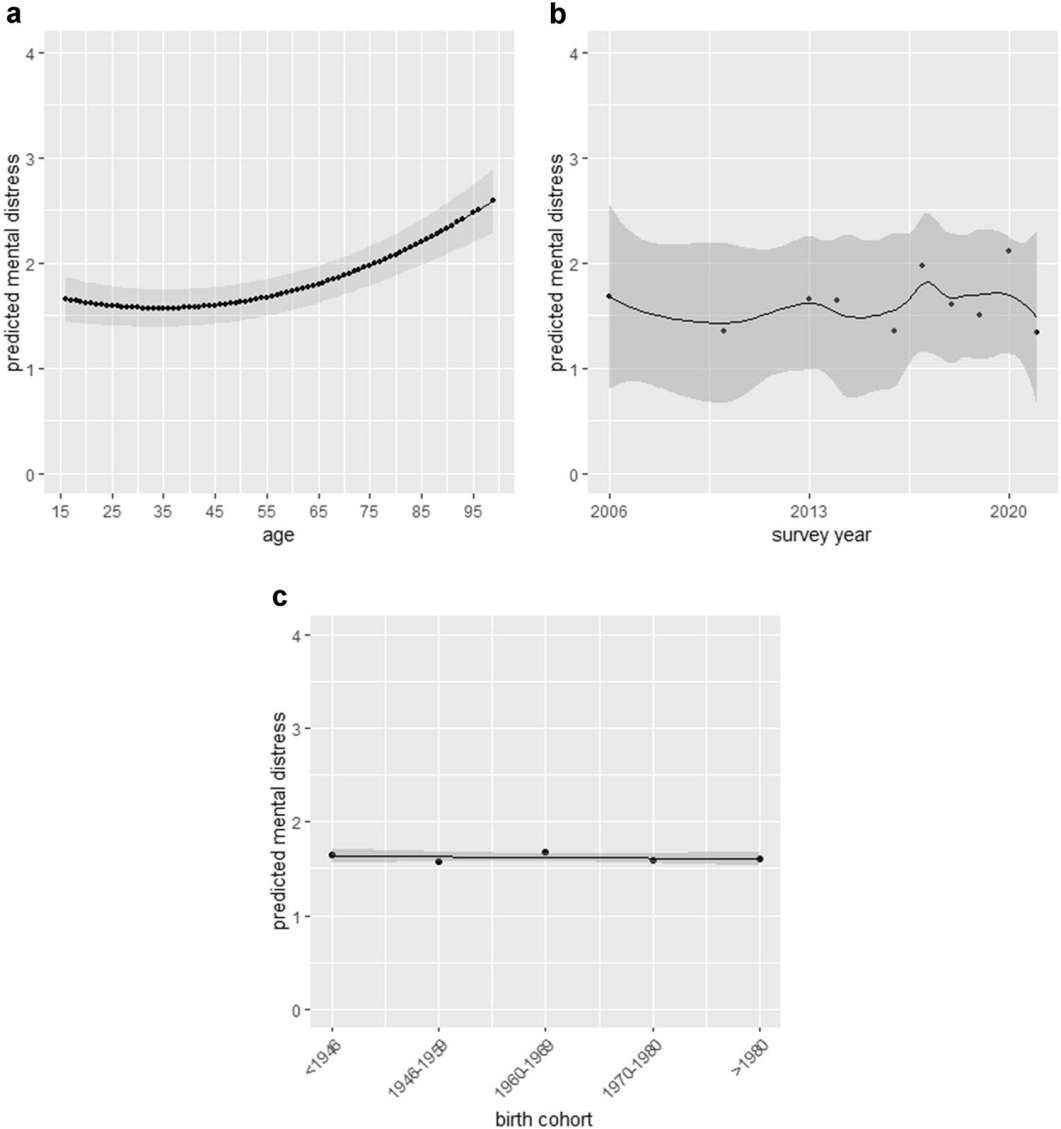
**a**–**c** Predicted age, age^2^, period, and cohort effects on mental distress without confounding variables. **a** Age and age^2^ effect, **b** period effect, **c** cohort effect. Period and cohort effects are based on random effects estimated from models, age effects are based on fixed-effects coefficients in models. The grey shade area represents the 90% confidence interval. In Figs. 2b and 2c, the points represent the predicted values per year and birth cohort, respectively, the trend line represents the total effect over years and birth cohorts

Model 1 of the HAPC analyses included only age, age^2^, period and cohort effects. Age had a significant negative effect on mental distress (− 0.017; 95% CI − 0.03, − 0.01), which suggested that after period and cohort effects were taken into consideration, the level of mental distress decreased by 1.7% with every one year increase in age. Figure 2a displays the combined effect of age and age^2^.

Predicted mental distress also varied by period and cohort, when controlling for the remaining two. The random effects variance components from Model 1 indicated smaller differences in mental distress outcomes by cohort than by period. The period effects showed strong fluctuations, especially in the last years. Predicted mental distress slightly decreased between 2006 and 2010, followed by a rebound in 2013–2014, but declining again thereafter. In 2017 and 2020, peak values of predicted mental distress were found. For details, see Fig. 2b. As to the cohort effect, predicted values of mental distress were highest in the oldest cohort, born before 1946. After that, mental distress decreases and remained constant over the cohorts, see Fig. 2c. These results are also displayed in Table 2 (s. Model 1). The explained variance for this model was 1.9%.

**Table 2:** Regression results of mental distress on gender, German region, and other control variables from hierarchical age-period-cohort models: Representative German Survey, 2006–2021

Fixed effects	Model 1	Model 2	Model 3	Model 4
	Coef. (95% CI)	Coef. (95% CI)	Coef. (95% CI)	Coef. (95% CI)
Intercept	1.698 (1.53; 1.87)***	1.480 (1.31; 1.65)***	2.268 (2.07; 2.47)***	1.617 (1.28; 1.95)***
Age	−0.017 (-0.03; −0.01)**	−0.016 (−0.03; −0.01)**	0.006 (−0.01; 0.02)	0.006 (−0.01; 0.02)
Age^2^	0.248 (0.14; 0.35)***	0.234 (0.13; 0.34)***	0.002 (−0.11; 0.11)	−0.003 (−0.11; 0.11)
Living area (ref. = west)				
East		0.059 (−0.01; 0.12)	−0.035 (−0.10; 0.03)	0.280 (0.07; 0.49)**
Gender (ref. = men)				
Women		0.381 (0.33; 0.43)***	0.303 (0.25; 0.36)***	0.552 (0.39; 0.72)***
Living with partner (ref. = yes)				
No			0.149 (0.09; 0.21)***	0.151 (0.09; 0.21)***
Household income (ref. = less than €1250)				
1250–2500			−0.834 (−0.91: −0.76)***	−0.833 (−0.91: −0.76)***
From 2500 onwards			−1.151 (−1.24: −1.07)***	−1.149 (−1.23: −1.06)***
Gender*living area (ref. = men*east Germany)				
Women*west Germany				− 0.207 (− 0.34; − 0.08)**

Random effects	Variance component	Variance component	Variance component	Variance component
Period effect (year)	0.069***	0.069***	0.074***	0.075***
Cohort effect (birth cohort)	0.003*	0.002*	0.003*	0.004*

Marginal *R*^2^/conditional *R*^2^	0.005/0.019	0.012/0.027	0.049/0.065	0.050/0.066

(1) *p < 0.05, **p < 0.01, ***p < 0.001, two-tailed test; (2) marginal *R*^2^ describes the proportion of variance explained by the fixed factors alone, conditional *R*^2^ describes the proportion of variance explained by both the fixed and random effects; (3) significance of random period and cohort effects was tested by comparing the fit of models without these terms (i.e., models with A + C and A + P) with the fit of the complete model (A + P + C), *p* values were obtained using the chi-squared distribution test; (4) continuous predictors were centered around the grand mean, reported estimates are standardized

To address possible biases of our results due to the widths of the cohort categories, we compared our results to results from alternative models in which the cohort was divided into several other categories. These alternative models did not significantly change the data fit and did not change the significance of the age and period effect. Only the cohort effect disappeared in one of the alternative models but remained significant in all other model variants. To test for linear effects of cohort and period, we compared our results to results from models in which cohort or period were modelled as solely fixed or both fixed and random effects. We observed a slightly better fit only for the model with a linear period effect and with cohort modelled as a random effect. However, additional tests did not indicate a significantly better fit for the data.

Models 2 through 4 investigated the effects of gender and German region on mental distress when controlling for age, age^2^, period, and cohort (s. Table 2). Age and age^2^ became insignificant when sociodemographic and socioeconomic control variables were included. The negative effect of age on mental distress thus disappeared. Furthermore, period and cohort differences slightly increased, indicating that gender, German region and other control variables did not explain the period and cohort effects.

**Fig. 3:**
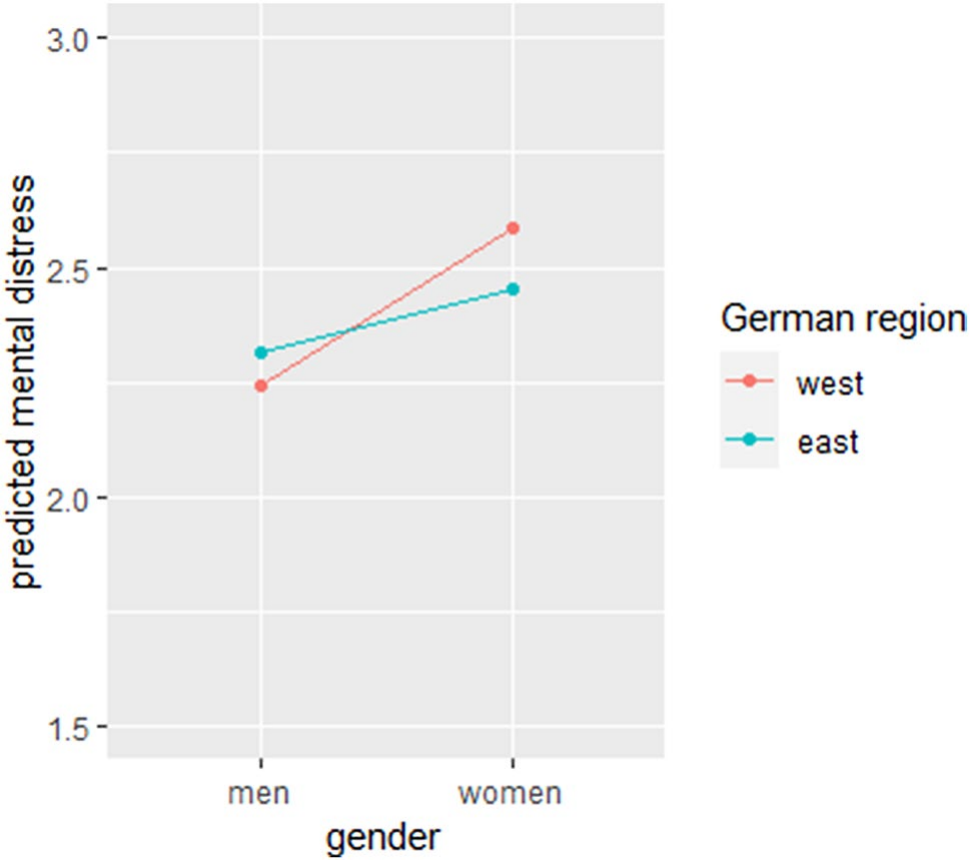
Interaction plot for gender and German region based on Model 4 of the HAPC analyses

In Model 2, significant differences for gender were found. Women reported 38.1% higher mental distress than men. However, no significant effect was found for the German region; no differences in reported mental distress were found between individuals living in East and West Germany. These findings remained when including socioeconomic and sociodemographic control variables (living with a partner and household income), as reported in Model 3. Respondents not living with a partner reported higher mental distress compared to respondents living with a partner. In addition, higher household income was associated with a decrease in mental distress. In the last model (Model 4), the included interaction term for gender and German region was found to be significant, indicating that women in West Germany reported significantly higher mental distress compared to women in East Germany. For men it is the other way around, men in East Germany report slightly higher levels of mental distress compared to men in West Germany. Women reported the highest values in both East and West Germany. This interaction is displayed in Fig. 3. The explained variance in this last model was 6.6%.

## Discussion

Findings on the mental health of residents from former Eastern and Western Germany have been contradictory. Analyzing repeated cross-sectional data from representative German surveys spanning 15 years, this study used HAPC models to disentangle age, time period and birth cohort effects on mental distress while testing for gender and regional effects (i.e. former Eastern and Western German states).

Findings revealed significant period effects. Research has shown that global public health is closely linked to political, economic, and social determinants [65]. In this study, peak values for mental distress were found in the years 2017 and 2020 for both women and men and in East and West Germany. Important political events can affect mental health in general and serve as an explanation for these peaks.

For the increased level of mental distress in 2017, one explanation can be found in several political and demographic upheavals taking place in the previous years. In a referendum in 2016, the United Kingdom decided to leave European Union. Furthermore, in November 2016 Donald Trump was elected president of the United States of America. These two events exemplify a wave of right-wing anti-globalization politics, which has risen in much of the Western world [65]. Shortly thereafter, in 2017, elections in three west European countries (France, the Netherlands, and Germany) took place and one of the main campaign issues was the alliance with the European Union. The discussions about this topic destabilized democratic cohesion, with antagonistic groups in society forming around this issue, further paving the way for right-wing parties such as the German *Alternative für Deutschland* [66]. These political movements were partly set in motion through the so-called “refugee crisis” in 2015 und 2016. In those years, large numbers of refugees from countries such as Syria, Iraq, Afghanistan, and Somalia applied for asylum in European countries which was shown to destabilize democracies in Europe [67] and which strongly contributed to the success of the radical right and right-wing populist parties [68]. This could have caused an increase in mental distress, since countries with a liberal democratic political system report on average more positive results on the population’s physical and mental health indicators [69]. Furthermore, due to the perceived threat associated with the “refugee crisis” and democratic instability, quarrels and protests increased. A systematic review from 2020 revealed compelling evidence that protests, also nonviolent, can be associated with adverse mental health outcomes, especially major depression [70].

The second peak in mental distress was found in 2020, which is likely to be caused by the COVID-19 pandemic and its first social lockdown restrictions. Literature has shown that this pandemic increased psychological health problems in Germany as in other countries. A systematic review and meta-analysis revealed that especially the prevalence of depression, anxiety, and distress increased during the pandemic [71]. A meta-analysis examining longitudinal cohort studies showed a small but significant increase in mental health symptoms early in the pandemic [72]. The survey included in this study took place at a similar time. However, effects for e.g. anxiety (but not depression) disappeared by mid-2020 and were comparable to pre-pandemic levels [72]. Furthermore, a large British study examining anxiety and depression symptoms found a decrease in symptoms during the first 20 weeks following the initial lockdown [73] Findings of this study came from an online panel and therefore could be biased [74, 75]. In German population surveys an increase in scores for depression and loneliness during the COVID-19 pandemic compared to scores in 2018 was found [76, 77], even though certain parts of the population (e.g. women, young people, high-risk of poverty) were affected most strongly [77, 78].

In addition to a period effect, a significant cohort effect was found. Mental distress was highest in the oldest birth cohort, born before 1946. This cohort experienced WW II and the hardships of the post-war era. The elderly have experienced higher lifetime trauma exposure and PTSD prevalence than younger persons [79–81]. Hence, this group may still suffer from mental health problems related to the traumatic WWII experience 50 years after the end of the war [82]. Traumatic events, especially war related, are highly connected to depressive symptoms [83, 84]. Furthermore, people from the birth cohorts 1946–1959 and 1960–1969 showed lower mental distress compared to people from the oldest birth cohort. However, they reported higher mental distress compared to people from the youngest two cohorts. A possible explanation for this could be experiencing the negative consequences of the transformation of the system after Germany was re-united, i.e., unemployment [85], or other economic and social differences [19], this applies above all to the former East German population.

Age in itself did not affect mental distress when cohort- and period effects were considered. Age effects found in other studies could therefore be merely a result of cohort or period effects. Another explanation could be found in the measure of mental distress in this study. PHQ-4 measures mental distress based on core depressive and anxiety symptoms. Previous research revealed depression to be less prevalent among older adults [7]. However, generalized anxiety disorder was shown to be higher among older age groups compared to younger age groups [86]. Therefore, the insignificant effect of age in this study could be caused by the different directions of the effects within our outcome variable.

Women reported significantly higher mental distress than men, which is in line with previous studies reporting more internalizing disorders (e.g., depression and anxiety) for women [87, 88]. This was consistent over the survey years, also in times of crisis. Other studies also confirmed this, e.g., women reporting higher mental distress during the COVID-19 pandemic than men [89, 90]. Unlike previous studies [27, 91], no significant difference between East- and West Germany in mental distress was found. Therefore, the period and cohort effect seems to play a role in the East-West differences. Interestingly, when combining gender and the German region, a significant interaction effect was present, revealing women in West Germany reported more mental distress compared to women in East Germany, whereas it was the other way around for men.

### Strength and limitations

This study is the first in Germany to examine age, period, and cohort effects in mental distress for a time period of 15 years including gender and German region. By applying HAPC models, we identified and separated age, period, and cohort effects. Furthermore, it is the first study to combine HAPC models with the generally untested assumption of measurement invariance in age, period, and cohort studies.

Yet, several limitations need to be considered when interpreting these results. Although the HAPC model is currently often applied as an approach to examine age, period, and cohort effects simultaneously, the discussion regarding the appropriate way to analyze such effects remains vivid, as strong assumptions have to be made about the nature of the effects [5]. Moreover, simulation studies revealed an underestimation of cohort effects when using the HAPC method [92]. Robustness checks are one way of addressing these issues, but future studies should consider other APC techniques to validate the results, e.g., the newly developed age-period-cohort interaction model [93]. Furthermore, the total time range covered in this study is still on the low end. Also, the number of years between the individual time points differ. In contrary to most APC studies, we used repeated cross-sectional sample survey data, which only simulates actual longitudinal data. Therefore, our findings do not provide insights into the possible causal effects for observed time trends. With regard to measurements, the classification of birth cohorts is partly theoretically and partly methodologically based, since a substantial amount of respondents is required in each cohort. We performed robustness tests using other cohort groups, which did not change our results. Lastly, we measured self-reported mental distress using the PHQ-4 scale. The PHQ-4 scale includes measured for symptoms of depression and anxiety, but results could differ from studies using solely depression or anxiety symptoms as an outcome as well as from studies that are based on diagnosed psychological disorders.

## Conclusion

The present work showed that the empirical analysis of factors associated with mental distress benefits from a multi-layered approach that differentiates risk and protective factors at different levels. Along these lines, the use of HAPC analyses yielded new insights: While results highlighted relevant period and cohort effects, no influence of age was found after sociodemographic and socioeconomic covariates were considered. Peak values for mental distress were found in the years 2017 and 2020. Further, mental distress was highest in the oldest birth cohort, born before 1946 and lowest in the youngest two birth cohorts. Finally, results of an interaction term between gender and region revealed higher mental distress for men in East Germany compared to men in West Germany while women reported the highest values in both East and West Germany.

These findings indicate that major crises that affect society as a whole influence the mental health of a population. Thus, mental distress is not only shaped by fixed characteristics of a person but also by structural societal factors. Therefore, effective prevention and intervention strategies must acknowledge structural differences. This could for example be done by investing in economic and political stability to reduce the hardships associated with major crises. Such an approach may be a beneficial extension of existing approaches for vulnerable groups within a society in addition to offering mental health support in the form of individual psychotherapy.

### Supplementary Information

Below is the link to the electronic supplementary material.

Supplementary file1 (PDF 159 KB)

## Data Availability

The datasets generated during and/or analysed during the current study are available from the corresponding author on reasonable request.
