# Supplementary material for: Gender differences in the prevalence of mental distress in East and West Germany over time: a hierarchical age-period-cohort analysis, 2006–2021
Source: Soc Psychiatry Psychiatr Epidemiol. 2023 Apr 11;59(2):315–28. doi: 10.1007/s00127-023-02479-z (PMC10089379; doi:10.1007/s00127-023-02479-z)
Supplement: Supplementary file 1 — Supplementary file1 (PDF 159 KB) [file 127_2023_2479_MOESM1_ESM.pdf]

Supplementary Table S1. Response rate for all included surveys.

Survey year	Recruitment in/between	initially selected	response rate	final sample	ethics approval number
2006	July 2006	8106	62.1	5036	
2010	January 2010	4069	61.5	2520	
2013	June and July 2013	4360	57.5	2508	050/13-11032013
2014	February and March 2014	4607	54.8	2527	063-14-10032014
2016	January-March 2016	4830	52.3	2524	452-15-21122015
2017	November 2017-January 2018 *(80% in 2017)*	5160	49.1	2531	418/17-ek
2018	Mai-July 2018	5418	46.4	2516	132/18-ek
2019	Mai-July 2019	5393	46.9	2531	145/19-ek
2020	April-June 2020	5418	47.3	2503	043/20-ek
2021	December 2020-February 2021 *(85% in 2021)*	5902	40.0	2519	474/20-ek

Note: Random-Route-procedure to select participants (using Kish-Selection-Grid to select random household and target person). Addresses no one was home after multiple visits from interviewer, persons refusing to participate or persons unable to participate (illness, vacation and so on) or breaking off the interview lead to final sample.

Supplementary Table S2a. Mental distress over time for the overall sample and stratified by gender and region.

	Total	Gender			Region		
		men	women	*p*-value	west	east	*p*-value
2006	1.76±2.06	1.57±1.97	1.93±2.12	<.001	1.69±1.99	2.04±2.29	<.001
2010	1.45±2.15	1.32±2.12	1.56±2.17	<.01	1.33±2.05	1.91±2.46	<.001
2013	1.74±2.29	1.46±2.09	2.00±2.42	<.001	1.71±2.25	1.87±2.43	.200
2014	1.71±2.18	1.50±2.07	1.90±2.25	<.001	1.73±2.20	1.63±2.05	.333
2016	1.42±2.18	1.21±2.01	1.59±2.31	<.001	1.36±2.18	1.64±2.20	<.05
2017	2.05±2.42	1.73±2.19	2.31±2.57	<.001	2.08±2.40	1.95±2.51	.300
2018	1.67±2.21	1.54±2.19	1.77±2.22	<.05	1.66±2.22	1.70±2.19	.698
2019	1.57±2.13	1.38±2.02	1.73±2.20	<.001	1.66±2.17	1.21±1.92	<.001
2020	2.18±2.31	1.91±2.17	2.41±2.40	<.001	2.17±2.30	2.21±2.34	.721
2021	1.41±2.10	1.26±1.99	1.55±2.18	<.001	1.46±2.09	1.22±2.10	<.05

Note: Reported are the mean value with standard deviation (M±SD).

Supplementary Table S2b. Mental distress across age groups for the overall sample and stratified by gender and region.

	Total	Gender			Region		
		men	women	*p*-value	west	east	*p*-value
<25 years	1.62±2.16	1.32±1.94	1.90±2.32	<.001	1.62±2.18	1.59±2.08	.777
25-34 years	1.58±2.12	1.31±1.92	1.82±2.26	<.001	1.59±2.12	1.54±2.16	.565
35-44 years	1.55±2.14	1.36±1.98	1.71±2.25	<.001	1.54±2.11	1.64±2.27	.258
45-54 years	1.73±2.20	1.62±2.16	1.81±2.23	<.010	1.71±2.16	1.81±2.36	.240
55-64 years	1.78±2.28	1.66±2.25	1.88±2.29	<.010	1.75±2.25	1.87±2.36	.187
65-74 years	1.64±2.11	1.41±1.98	1.86±2.21	<.001	1.66±2.15	1.59±2.01	.357
>74 years	2.24±2.47	1.90±2.33	2.46±2.54	<.001	2.17±2.39	2.42±2.67	.055

Note: Reported are the mean value with standard deviation (M±SD).

Supplementary Table S2c. Mental distress across cohorts for the overall sample and stratified by gender and region.

	Total	Gender			Region		
		men	women	*p*-value	west	east	*p*-value
<1946	1.99±2.33	1.66±2.18	2.26±2.41	<.001	1.96±2.29	2.10±2.42	.078
1946-1959	1.70±2.18	1.59±2.12	1.81±2.22	<.001	1.68±2.17	1.78±2.21	.168
1960-1969	1.71±2.22	1.57±2.18	1.83±2.24	<.001	1.69±2.18	1.81±2.37	.140
1970-1980	1.54±2.10	1.37±1.95	1.69±2.21	<.001	1.54±2.08	1.54±2.20	.935
>1980	1.60±2.18	1.32±1.96	1.86±2.33	<.001	1.62±2.19	1.54±2.13	.241

Note: Reported are the mean value with standard deviation (M±SD).

Supplementary Table S3. Multi-group factor analyses for survey year, cohort and age groups.

	χ^2	df	$\Delta\chi 2$	p	CFI	Δ CFI	SRMR
Survey year (period)							
Configural	982.742	20	---	<.001	0.981	---	0.020
Metric	1246.533	47	263.791	<.001	0.976	-0.005	0.036
Scalar	1592.580	74	346.047	<.001	0.970	-0.006	0.041
Generations (birth cohorts)							
Configural	911.343	10	---	<.001	0.982	---	0.020
Metric	956.291	22	44.948	<.001	0.981	-0.001	0.023
Scalar	992.348	34	36.057	<.001	0.981	0.000	0.024
Age groups							
Configural	886.670	14	---	<.001	0.982	---	0.019
Metric	923.605	32	36.935	<.001	0.982	0.000	0.022
Scalar	970.217	50	46.612	<.001	0.981	-0.001	0.023

Note: df = degrees of freedom; CFI = Comparative Fit Index; TLI =Tucker-Lewis Index, SRMR=Standardized Root Mean Square.
